# Novel Findings into AIRE Genetics and Functioning: Clinical Implications

**DOI:** 10.3389/fped.2016.00086

**Published:** 2016-08-22

**Authors:** Lucia De Martino, Donatella Capalbo, Nicola Improda, Paola Lorello, Carla Ungaro, Raffaella Di Mase, Emilia Cirillo, Claudio Pignata, Mariacarolina Salerno

**Affiliations:** ^1^Pediatric Section, Department of Translational Medical Sciences, Federico II University, Naples, Italy; ^2^Department of Pediatrics, Federico II University, Naples, Italy

**Keywords:** APECED, autoimmune disease, diagnosis, AIRE, mutations

## Abstract

Autoimmune polyendocrinopathy candidiasis ectodermal dystrophy (APECED), formerly known as autoimmune polyendocrine syndrome type 1, is a paradigm of a monogenic autoimmune disease caused by mutations of a gene, named autoimmune regulator (*AIRE*). AIRE acts as a transcription regulator that promotes immunological central tolerance by inducing the ectopic thymic expression of many tissue-specific antigens. Although the syndrome is a monogenic disease, it is characterized by a wide variability of the clinical expression with no significant correlation between genotype and phenotype. Indeed, many aspects regarding the exact role of AIRE and APECED pathogenesis still remain unraveled. In the last decades, several studies in APECED and in its mouse experimental counterpart have revealed new insights on how immune system learns self-tolerance. Moreover, novel interesting findings have extended our understanding of AIRE’s function and regulation thus improving our knowledge on the pathogenesis of APECED. In this review, we will summarize recent novelties on molecular mechanisms underlying the development of APECED and their clinical implications.

## Introduction

Autoimmune polyendocrinopathy candidiasis ectodermal dystrophy (APECED), formerly known as autoimmune polyendocrine syndrome type 1 (APS-1), is a rare disease caused by mutations of the autoimmune regulator (AIRE) which acts as a transcription regulator that promotes immunological central tolerance (1).

Autoimmune polyendocrinopathy candidiasis ectodermal dystrophy represents a paradigm of genetically determined systemic autoimmunity. However, the great variability that characterizes APECED, irrespectively of AIRE genotype, implies that additional factors modulate the clinical expression of the disease.

Recent advances on how AIRE affects immunological tolerance and is linked to organ-specific autoimmunity have improved our understanding on the pathogenesis and the wide variability of clinical expression of APECED.

In this review, we will summarize new insights into AIRE genetics and functioning and its implications on APECED phenotype.

## New Insights into Aire Function

Autoimmune regulator is known to exert a crucial role in central tolerance and negative selection of autoreactive T cells (1). The induction of central tolerance is an intricate process that occurs within the thymus where immature T lymphocytes are “committed” to become mature cells able to respond to a huge number of foreign antigens, but preventing autoimmune reactions. Medullary thymic epithelial cells (mTECs) have a primary role in the negative selection and, in this context, AIRE acts as a crucial transcriptional regulator. In mTECs, AIRE induces promiscuous gene expression (pGE) of tissue-specific antigens (TSAs), which are, then, presented to maturing T cells. Autoreactive T cells that recognize these TSAs with high affinity undergo negative selection through their apoptosis or, alternatively, regulatory T cells (Treg) are generated in order to prevent autoimmunity (2, 3).

Autoimmune regulator gene encodes a 545 amino acid protein with a molecular weight of 58 kDa (1). Starting from the amino terminus, AIRE is composed of a caspase recruitment domain (CARD)/homogeneously staining region (HSR), nuclear localization sequences (NLS), a SAND (Sp100, AIRE NucP41/75, and DEAF) domain, two planthomeodomain (PHD) zinc fingers, a proline-rich region (PRR), and four LXXLL motifs (where L stays for leucine) distributed among the domains (4). The CARD/HSR is involved in the process of AIRE homomultimerization and seems also to anchor AIRE to the chromatin (4, 5). The NLS has a stretch of basic amino acids at positions 131–133 important for nuclear import (4). The SAND domain does not have a distinct DNA-binding motif, but it is involved in promoting a protein–protein interaction with a transcriptional repressive complex (6). The two AIRE PHD fingers form a structural system for the recruitment of chromatin-related proteins and are engaged in AIRE transcriptional activity (7–9). LXXLL motif and PRR are implicated in promoting gene transcription (4).

Autoimmune regulator has a strict spatiotemporal regulation, being ubiquitously transcribed during the earliest stages of embryogenesis, and then restricted to thymic cells (mTECs and B cells) and extra thymic hematopoietic stem cells that may have a role in CD4 tolerization (10, 11).

At the transcriptional level, the expression of AIRE in mTECs and peripheral lymphoid organs is regulated by receptor activator of nuclear factor κB (RANK) signaling and therefore by nuclear factor κB (NF-κB)-induced transcription through an upstream conserved non-coding sequences (CNSs) of the *Aire* gene containing NF-κB-binding sites (12, 13). In addition, post-transcriptional mechanisms seem to modulate AIRE expression. A dioxygenase that catalyzes lysyl hydroxylation of splicing regulatory proteins (Jmjd6) is critical for AIRE expression. In fact, the intron 2 of *Aire* gene is not effectively spliced out in the absence of Jmjd6, resulting in marked reduction of mature Aire protein in mTECs and spontaneous development of multi-organ autoimmunity in mice (14).

**Figure 1:**
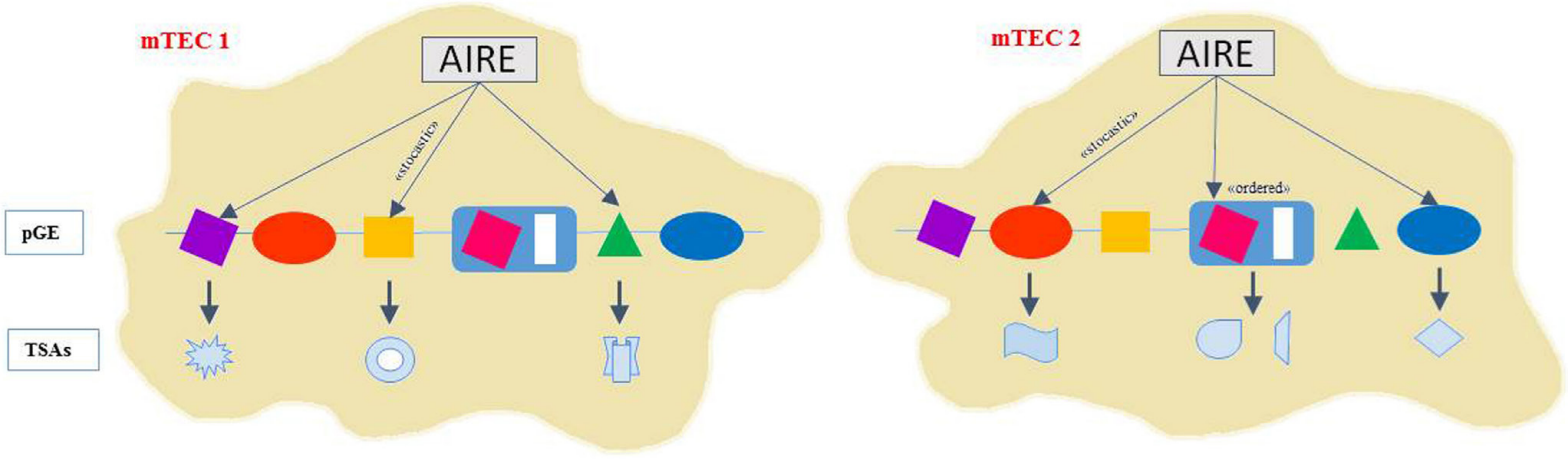
**AIRE controls gene expression with ordered stochasticity**. AIRE seems to regulate pGE in mTECs in an apparently stochastic manner. Thus, single mTECs would express TRAs of mixed tissue origin rather than emulating cell line age-affiliated patterns displaying the highest degree and diversity of pGE. Indeed, different sets of TSAs are expressed in mTECs but whether a particular AIRE-regulated TSA is expressed in a given mTEC seems to be highly probabilistic. The “ordered” TSA expression refers to the increased likelihood that a particular set of TSA genes will be coexpressed in an individual mTEC. Coexpressed gene loci tend to colocalize to the same nuclear subdomain and TSA subsets align along progressive differentiation stages within the mature mTEC subset.

The AIRE protein resides inside the nucleus, where it exhibits a speckled localization pattern (15). AIRE is a key regulator of TSA expression in mTECs and affects the transcription of thousands of TSA genes in a “stochastic” and “ordered” manner (16, 17). Indeed, a small percentage (1–3%) of the total number of mTECs expresses a particular TSA (18). Different sets of TSAs are regulated by AIRE within individual mTECs but whether a particular AIRE-regulated TSA is expressed in a given mTEC seems to be highly probabilistic (18, 19). Moreover, the “ordered” TSA expression refers to the increased likelihood that a particular set of TSA genes will be coexpressed in an individual mTEC (20) (Figure 1).

Autoimmune regulator acts in a very unusual way among transcription regulators, as it has no clear DNA-binding motif but seems to recognize genes that possess silenced chromatin states (6, 8, 9, 16). AIRE does not directly initiate TSA gene transcription, but it promotes TSA expression through the release of stalled RNA polymerase, RNA elongation, and splicing of target TSAs (15, 21). Moreover, AIRE binds to several partners that have the potential for post-translational protein modification, including the modification of AIRE itself and that seem to be critical for its biological function (22–24).

Recent insights on AIRE’s regulation come from experimental studies which suggest that estrogen induces epigenetic changes in the AIRE gene, leading to reduced AIRE expression under a threshold that increases susceptibility to autoimmune diseases (25).

In summary, induction of pGE by AIRE is dependent on a complex regulatory mechanism which has only been partially unraveled so far.

In addition to the key role exerted on pGE, AIRE seems also to be critical for thymic generation of Treg cells during the perinatal period (3, 26). However, on this issue, further work is needed (3).

Moreover, recently a new hypothesis on Aire functioning in tolerance has been postulated. Aire may enforce immune tolerance by ensuring that autoreactive T cells differentiate into the Treg cell lineage; dysregulation of this process results in the diversion of Treg cell-biased clonotypes into pathogenic conventional T cells (27).

Furthermore, AIRE has several functions that are independent of its promotion of TSA expression in mTECs such as immunoregulatory functions in extrathymic AIRE-expressing cells and thymic B cells (15, 28). Moreover, AIRE enhances negative selection by regulating the repertoire of thymic dendritic cells and promoting apoptosis of mTECs (29, 30).

Finally, it has been postulated that AIRE regulates thymic maturation and architecture, probably through the expression of microRNAs (15, 31–34).

In summary, although our knowledge has increased in recent years, we still lack a coherent model incorporating and explaining all the intricacies of AIRE and its role in the regulation of immunological tolerance.

## New Insights into Aire Mutations

In humans, AIRE, identified on chromosome 21q22.3 by positional cloning in 1997, consists of 14 exons spanning 11.9 kb of genomic DNA (15). Mutations in the AIRE gene result in the development of APECED, a rare autoimmune condition, but reported worldwide, with a higher prevalence in genetically isolated populations (1).

Nowadays, 101 APECED-causing mutations have been found throughout AIRE (http://www.hgmd.cf.ac.uk/ac/gene.php?gene=AIRE). These mutations include nonsense/missense mutations, deletions, or insertions and often abolish AIRE transcriptional activity or its localization to nuclear bodies (15, 35).

Despite its monogenic nature, APECED is characterized by a wide variability of the clinical expression and no strong genotype–phenotype correlation has been found among several populations (1, 35). Noteworthy, this lack is exemplified by the significant intrafamilial differences even between siblings carrying the same mutation, suggesting that disease-modifying genes, environmental factors, and immune system dynamics may play a role in modulating clinical expression of the syndrome (36, 37).

Autoimmune polyendocrinopathy candidiasis ectodermal dystrophy has been originally considered an autosomal–recessive disease, and most mutations were assumed to be inherited in an autosomal–recessive manner, except for one mutation in the SAND domain, p.G228W, which exerts a dominant inheritance pattern (38).

However, recent evidences highlight that also heterozygous mutations of the gene can be associated with increased susceptibility to autoimmune diseases or incomplete forms of APS-1 (39). Patients with atypical or incomplete manifestations of APECED or with other immune diseases carrying heterozygous mutations of AIRE have been also described (38, 40–49). Cervato et al. showed different AIRE mutations in heterozygous state in relatives of APECED patients with various degrees of autoimmune or non-autoimmune diseases, but none of which affected by one of the major components of APECED (50).

Recently, Oftedal et al. reported a group of novel monoallelic and dominant-negative AIRE mutations clustered within the first PHD1 zinc finger domain in patients with various degrees of autoimmunity (39). The PHD1 domain is critical for AIRE’s transcription–transactivation activity and mutations in this domain seems to affect the structure and thus the function of the entire AIRE tetramer. However, the significance of these monoallelic mutations is still unclear since the same alterations were found in varying autoimmune phenotypes, ranging from milder phenotypes of late-onset APECED to autoimmune polyglandular syndrome type 2 (APS-2) and isolated organ-specific autoimmunity following incomplete inheritance. A possible explanation is that AIRE tetramers still have some residual activity sufficient to ensure partial self-tolerance. Moreover, PHD1 mutations scanned in a public databases revealed an estimated frequency of about 0.0008, which is in the range of several autoimmune conditions that affect about 1 in 1,000 people, thus suggesting that mutations in AIRE might be more widespread in patients with autoimmunity than previously thought (39).

Moreover, Sparks et al. identified additional dominant-negative AIRE mutations associated with the modulation of insulin gene expression in thymus which is essential to induce either insulin tolerance or the development of insulin autoimmunity and type 1 diabetes (51).

## Apeced: From “Classical” to “Non-Classical” Phenotype

In the light of these new knowledges, the original classification of APECED as unique autosomal–recessive disease seems to be incomplete. Taking into account the huge spectrum of phenotypes related to AIRE mutations, Oftedal et al. interestingly proposed to differentiate APECED in two major forms: (1) “classical APECED,” characterized by recessive inheritance, presence of at least two of the three main components, and interferon (IFN) antibodies; and (2) “non-classical APECED,” characterized by dominant heterozygous mutations mainly in AIRE’s PHD1 zinc finger and a milder, less penetrant autoimmune phenotype (39) (Table 1).

**Table 1:** **APECED in “classical” and “non-classical” forms**.

	“Classical APECED”	“Non-classical APECED”
Inheritance	AR	AD
Mutation	Homozygous/compound heterozygous	Heterozygous
Phenotype	APECED (two of the three main components)	Various degrees of autoimmunity (from late-onset classical APECED or APS-2 to isolated organ-specific autoimmunity, i.e., vitamin B12 deficiency, pernicious anemia, vitiligo)
Onset	Childhood	Childhood/adulthood
Penetrance	Complete	Incomplete
IFN antibodies	Present	Variable

Classical diagnosis of APECED has been originally defined by the presence of two of the three most common features: chronic mucocutaneous candidiasis (CMC), chronic hypoparathyroidism (CH), and Addison’s disease (AD) (52).

Neutralizing autoantibodies against type 1 IFN (especially IFN-ω and IFN-α) have been found to strictly correlate with AIRE deficiency, thus leading to consider these autoantibodies as a precocious diagnostic tool for APECED and an additional diagnostic criteria for the diagnosis of APECED (52, 53). However, IFN autoantibodies seem to be less prevalent in the “non-classical” form, probably reflecting some residual AIRE function (39).

Molecular analysis of *Aire* may help to confirm the clinical diagnosis, especially in those cases with an atypical presentation.

Both “classical” and “non-classical” phenotypes are characterized by a wide heterogeneity in the severity and in the number of components among affected subjects with a wide variability even between siblings with the same genotype (39, 54).

Chronic mucocutaneous candidiasis is the first sign to appear followed by CH, before the age of 10 years, and later by adrenal insufficiency. However, a precise chronological order is not always present (52).

In addition to the classic triad (CMC, CH, and AD), the phenotype of APECED includes several autoimmune manifestations, which in some cases may also precede the classical triad (52).

The spectrum of endocrinopathies associated with APECED includes hypergonadotropic hypogonadism, type 1 diabetes (T1D), autoimmune thyroid diseases (ATD), growth hormone (GH) deficiency, and other pituitary defects (52).

The appearance of ectodermal abnormalities is also quite common including dental enamel hypoplasia, pitted nail dystrophy, and alopecia. Keratopathy, vitiligo, calcifications of the tympanic membranes, and periodic maculopapular, morbilliform, or urticarial rash with fever (52) are also included in the clinical spectrum of APECED.

Furthermore, gastrointestinal autoimmunity in APECED may lead to autoimmune gastritis, autoimmune hepatitis (AIH), intestinal disorders with chronic diarrhea alternating with obstipation, and cholelitiasis (54).

Asplenia, tubulointerstitial nephritis, interstistial lung disease (ILD), vasculitis, Sjogren’s syndrome, cutaneous vasculitis, hemolytic anemia, scleroderma, metaphyseal dysplasia, and celiac disease have also been reported in APECED (55–58). Recently, a diagnosis of APECED was established by performing whole exome sequencing in a patient with increased renal echogenicity on renal ultrasound (59).

Muscle disease, with very similar clinical features of progressive limb-girdle myopathy, is a rare component of APECED (60).

To date, two patients with APECED have been affected by encephalitis leading to a severe and life-threatening condition (61, 62).

The “non-classical” form of APECED has been suggested to be characterized by variable autoimmune phenotypes, ranging from late-onset APECED to different combinations of autoimmune manifestations (APS-2), isolated organ-specific autoimmunity or autoantibodies, but no signs of autoimmune disease within individuals who harbor monoallelic AIRE mutations. In particular, families with vitamin B12 deficiency, pernicious anemia, and/or vitiligo at early age have been found to carry heterozygous PHD1 mutations, although the clinical phenotype has been expanded when larger materials were investigated. Indeed, organ-specific autoimmunity in the heterozygous cases seems to present in milder form and incomplete penetrance with respect to classical (39). These observations open a new window on the possibility that mutation carriers have a risk for developing some degree of APECED or other form of polyendocrinopathy.

However, more research is needed to determine the contributions of such AIRE variants to autoimmune susceptibility, especially in kindreds with a strong family history of autoimmunity.

In either “classical” or “non-classical” form of APECED, early diagnosis and regular surveillance, including periodic evaluation of hormonal and biochemical parameters, are essential to allow the prevention of severe and life-threatening events (i.e., hypocalcemia, adrenal crisis), even in the absence of clinical symptoms (63).

## Old and New Autoantibodies

Autoimmune polyendocrinopathy candidiasis ectodermal dystrophy features multi-organ autoimmunity and autoantibody responses against target molecules with restricted tissue expression profiles. Consequently, autoantibody markers have acquired a central role in research and clinical diagnosis of APECED, providing a tool for diagnosis, and as predicting factor for the clinical course of the disease.

Chronic mucocutaneous candidiasis is a sign of the underlying immunodeficiency. Although the pathogenesis of CMC seems to be different from the all other autoimmune manifestations of the disease, an autoimmune pathogenesis for APECED-related CMC has also been proposed (64). APECED patients also develop high titer of neutralizing autoantibodies against IL-22, IL-17F, and IL-17A (64). Indeed, the neutralizing autoantibodies to Th17 cytokines or the impaired production of IL-22 and IL-17A seem to be associated with susceptibility to *Candida* infection (64).

The occurrence of endocrine manifestations is usually associated with a specific array of organ-specific autoantibodies. NATCH leucine-rich repeat protein 5 (NALP5) has been identified as the target for autoimmune attack in the parathyroid cells (65) in APECED. Autoantibodies specific for the steroidogenic enzymes (CYP21A2 and CYP17A1) and side chain cleavage enzyme (CYP11A1) are useful markers for the autoimmune destruction of the adrenal cortex even years before the clinical onset of the disease (5, 66). Autoantibodies to cytochrome CYP11A1 are associated with ovarian insufficiency (66). T1D is correlated with autoantibodies against insulin, IA-2 tyrosine phosphatase-like protein, and glutamic acid decarboxylase GAD65 (67).

Autoimmune hepatitis is characterized by the presence of autoantibodies specific for liver-expressed cytochromes CYP1A2 and CYP2A6 (67). Gastrointestinal symptoms have been associated with the presence of autoantibodies against tryptophan hydroxylase (TPH) (68). Enteroendocrine cells can also be the target of an autoimmune attack. Therefore, in some cases, the intestinal dysfunction might be viewed as an autoimmune endocrinopathy (69). Recently, circulating autoantibodies to Paneth cell-specific alpha 5 defensin and reduced numbers of Paneth cells in APECED patients have been reported and also associated with intestinal dysfunction (70).

Autoantibodies against TPH have been associated with alopecia, vitiligo, and enamel dysplasia and anti-SOX9/SOX10 antibodies with vitiligo (71, 72).

Autoantibodies directed against the potassium channel regulatory protein (KCNRG) and BPIFB1, found in epithelial cells of terminal bronchioles, have been suggested as a marker for pulmonary disease in APECED patients (64, 73).

The autoimmune nature of renal destruction has been confirmed by examining biopsy samples and by determining antiproximal tubular autoantibodies (74, 75). Furthermore, autoantibodies targeting kidney collecting ducts specific antigens [aquaporin 2 (AQP2) and two transcription factors regulating the aquaporin 2 promoter, namely homolog of the human homeobox B7 (HOXB7) and NF of activated T cells 5 (NFAT5)], have been recently identified in APECED patients affected with tubulointerstitial nephritis (76).

Recently, B cell response against a panel of over 9,000 human proteins has enabled to have a detailed profiling of known autoantigens and to identify novel immune targets in APECED. As for the lack of genotype–phenotype relationship, it has been shown that AIRE genotype did not appear to be an important determinant of autoantibody expression. Moreover, two novel gonadal autoantigens, melanoma antigen family B 2 (MAGEB2) and protein disulfide isomerase-like testis (PDILT), have been identified that potentially could contribute to infertility in male and female patients with APECED (77). Another mechanism proposed to explain subfertility in males with APECED is the presence of autoantibodies against the prostatic antigen transglutaminase 4 (TGM4), causing prostatitis, and possible abnormal sperm maturation (78).

Neutralizing autoantibodies specific for type I IFNs discovered in 2006 by Meager et al. (79) are hallmark of the “classical” APECED and are detectable in AIRE-deficient children as early as a few months of age, before the appearance of clinical symptoms or organ-specific autoantibodies (79). Autoantibodies against IFN seems to be less prevalent in “non-classical” APECED, probably reflecting some residual AIRE function (39).

In conclusion, we should take into account that the autoimmune response in APECED appears orders of magnitude more limited than could be expected. There is a great discrepancy between the number of AIRE-controlled genes (around 4,000) and the number of detected autoantigens in APECED (around 20). Several explanations must be considered. First, it could be that only a subset of self-antigens is able to activate autoimmune responses. Moreover, the peripheral tolerance mechanisms may provide additional filters for the development of autoimmunity. Finally, it has been observed that autoimmunity in APECED preferentially targets molecules with restricted tissue expression profiles.

## New Insights into Aire Genetics and Functioning: Clinical Implications

The genetic basis of autoimmunity is a complex problem. The main lesson from recent evidence is that the mutations of AIRE can lead to various degrees of clinical autoimmunity, ranging from “classical” APECED to specific autoimmune conditions, which had not been previously mined for genetically determined conditions. Therefore, partial alterations of AIRE could play a role in common autoimmune disease; however, to measure the penetrance and the relative risk conferred by pathogenic AIRE mutations in its monoallelic variants, it will be necessary to sequence *Aire* in large cohorts of healthy individuals and autoimmune patients and to characterize experimentally in-depth all mutant alleles.

Furthermore, in the last decade, knowledge of AIRE’s function and regulation has been significantly expanded leading to the identification of several partners and regulators of AIRE. Taken together, these molecular insights open new perspectives in understanding the phenotypic variability related to AIRE mutations and might provide interesting targets for novel therapeutic approach.

Indeed, an unanimously accepted effective therapy for APECED is not currently available. The use of immunosuppressive treatment in this category of patients may lead to a transient immunodeficiency with the risk to worsen their CMC and seems not able to stop the progression of all APECED manifestations (80). Thus, the management is mainly based on the care of each individual component and is mainly characterized by substitutive treatments for hormone deficiencies and immunomodulators have been only used in selected severe phenotypes (80, 81).

Although thymic transplantation has been proven useful in the treatment of differentiative thymic disorder (82), no data are available on this intervention on alterations of the thymic negative selection process. Thymic compartment can be targeted to modulate immune tolerance, for example, by enhancing AIRE expression, promoting deletion of self-reactive T cells and enhancing positive Treg cell selection, or inducing differentiation of TECs from pluripotent stem cells, offering new exciting possibility in therapeutic manipulation (15).

In conclusions, the new insights in the biology of AIRE and its control in immune tolerance offer exciting possibilities for the exploration of diagnostic and therapeutic strategies that would benefit APECED patients.

## Author Contributions

All the authors contributed equally to this work.

## Conflict of Interest Statement

The authors declare that the research was conducted in the absence of any commercial or financial relationships that could be construed as a potential conflict of interest.
